# Good Fences Make Good Neighbors: Adjacent Honey Bee Colonies Locally Partition Their Foraging Across Landscapes

**DOI:** 10.1002/ece3.71401

**Published:** 2025-05-15

**Authors:** Bradley D. Ohlinger, Margaret J. Couvillon, Laurence W. Carstensen, Roger Schürch

**Affiliations:** ^1^ Odum School of Ecology University of Georgia Athens Georgia USA; ^2^ Department of Entomology Virginia Tech Blacksburg Virginia USA

**Keywords:** intraspecific competition, optimal foraging theory, resource partitioning, waggle dance

## Abstract

Optimal foraging theory (OFT) predicts that animals employ foraging strategies that maximize a particular currency, such as net energetic efficiency, to meet their nutritional demands. Two nonexclusive patterns that arise from OFT are convergence on high‐quality resources and resource partitioning. Honey bees make collective decisions by integrating their individual foraging with social recruitment behaviors: returning foragers communicate the approximate vector to high‐quality resources using waggle dances. Because we can eavesdrop on their communications, waggle dance decoding is a valuable tool for exploring OFT predictions as it allows us to map how honey bees use landscapes. In this study, we analyzed 8049 dances from colocalized colonies across three landscapes to investigate whether neighboring colonies forage by not partitioning patches (i.e., converging their food collection on the same patches), by partitioning at the landscape level, or by partitioning at the local level. To differentiate between these three possible scenarios, we examined three metrics: (1) interdance distances between and within colonies; (2) k‐nearest neighbors; and (3) k‐means clustering. We observed no difference in the distances between dances performed by bees from the same colony compared to those from different colonies. Also, we found at each of the three field sites that dances from the same colony were not more likely to appear as close neighbors to each other. Finally, k‐means cluster analysis demonstrates that dance locations advertised by the same colony aggregated nonrandomly in the three sites, where dances from the same colony comprised a significant majority of dances within k‐means clusters and 62% of clusters consisted entirely of dances from a single colony. Together, these results support a foraging scenario where honey bees partition their foraging, but at the local level. This strategy may help limit intercolony foraging competition.

## Graphical abstract

In this article, we explore optimal foraging theory predictions for competing honey bee colonies by quantifying the relative spatial foraging patterns of 8049 waggle dances from colocalized colonies across three landscapes. Our results demonstrate that neighboring colonies distribute their foraging widely at the landscape scale, while partitioning resources at the local scale. These findings suggest that honey bees employ a foraging strategy that simultaneously exploits available resources and limits inter‐colony competition.

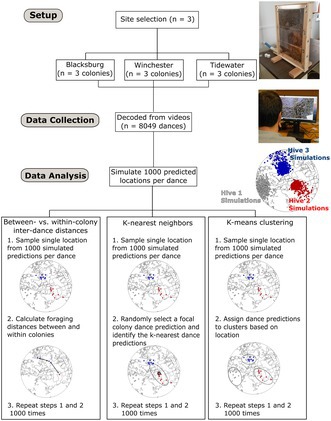

## 1 Introduction

Foraging is necessary for animals to acquire the energy and nutrition needed for development, survival, and reproduction. Animals collect food in complex environments, comprised of multiple foraging options that may vary in their abundance (Lanza et al. 1995; Bagchi et al. 2003; Lucas et al. 2017) and quality (Chalcoff et al. 2006; Razeng and Watson 2015; Pamminger et al. 2019; Quinlan et al. 2021; Venjakob et al. 2022). As a result, foragers must reliably and efficiently decide which resources to collect and how to distribute their efforts across foraging options (T. D. Seeley 1986; Seeley et al. 1991). To accomplish this task, animals integrate a suite of sensory (Farina et al. 2005, 2012; Chittka and Raine 2006; Rusch et al. 2016) and cognitive (Laverty 1994; Gumbert 2000; Abramson et al. 2013, 2016; Howard et al. 2018) capabilities to identify resources in the environment and assess their nutritional value against the cost of collection (T. D. Seeley 1994; Ohlinger et al. 2022).

Optimal foraging theory (OFT) proposes that organisms forage to maximize a particular nutritional/energetic currency, such as the net rate of energetic uptake, as seen in starlings (Bautista et al. 2001), or net energetic efficiency, as seen in honey bees (Schmid‐Hempel et al. 1985; T. D. Seeley 1994). This prediction has been tested by decades of research investigating foraging behavior through the lens of OFT across various taxa with distinct diets, social nesting strategies, and habitats (Ding et al. 2020; Roeder et al. 2020; Keesing 2021). Additionally, OFT research suggests that we can reliably predict foraging patterns if we understand the currencies of fitness used and the characteristics of the foraging landscape (Pyke et al. 1977; Pyke 1984; Pyke and Starr 2021).

Honey bees are highly efficient social foragers that employ both individual‐ and colony‐level adaptations to maximally meet their nutritional demands (Seeley et al. 1991; T. D. Seeley 1994; T. Seeley 1995). Interestingly, the foraging process often begins with random searching for available food sources via scouting (T. D. Seeley 1983; Biesmeijer and Seeley 2005) and then ends with selective exploitation of a few high‐quality resources via individual foraging and recruitment (Seeley et al. 1991; T. D. Seeley 1994; T. Seeley 1995; Nürnberger et al. 2019; Shackleton et al. 2023). Selective exploitation occurs in this way: after finding a high‐quality resource, a foraging bee communicates its approximate location to her nestmates using the waggle dance (von Frisch 1967; T. Seeley 1995). The dancing bee will perform a repeated figure‐eight movement consisting of two discrete components: a waggle phase, during which she shakes her body and runs linearly across the comb, and a return phase, during which she turns back around to perform another waggle phase. The waggle phase's direction relative to the vertical comb communicates the direction from the colony to the food, relative to the solar azimuth, and the duration of the waggle phase communicates the distance to the food (von Frisch 1967; Couvillon 2012). By following waggle dances, individual foragers can find the advertised food in the landscape (von Frisch 1967; Gould 1975) and then focus their foraging on resources that meet their colony's current nutritional needs (T. D. Seeley 1989, 1995; Camazine 1993; Dreller et al. 1999; Dreller and Tarpy 2000). Because we can observe this recruitment behavior, waggle dance decoding has been developed to extract the information to map honey bee foraging (Couvillon et al. 2012; Schürch et al. 2013, 2019). This emerging method has been used to investigate honey bee foraging in seminatural (Visscher and Seeley 1982; Steffan‐Dewenter and Kuhn 2003), agricultural (Garbuzov, Couvillon, et al. 2015; Balfour and Ratnieks 2017; Lin et al. 2022; Silliman et al. 2022; Steele et al. 2022; Ohlinger et al. 2024), and urban landscapes (Waddington et al. 1994; Garbuzov, Schürch et al. 2015; Sponsler et al. 2017). As a result, waggle dance decoding is now considered an effective method for investigating the spatial patterns of honey bee foraging (Couvillon and Ratnieks 2015).

Our current theoretical understanding, inspired by OFT, predicts that colocalized, or neighboring, colonies should converge on the same high‐quality patches within their foraging range if patches provide ad libitum food. In other words, they should not partition the landscape into colony‐specific foraging clusters. On the other hand, OFT predicts that *limited* resources may be *partitioned*, and we do indeed know that competition between conspecifics often leads to resource partitioning in both solitary animals, such as the common raven (Marzlufi and Heinrich 1991) and the Iberian lynx (López‐Bao et al. 2011), and highly social animals, like the red imported fire ant (Wilson et al. 1971) and termites (Haverty et al. 1975). Interestingly, previous research (Table 1) has suggested that honey bee foragers from neighboring colonies may, in fact, partition the landscape (Waddington et al. 1994; Beekman et al. 2004). However, these data are limited in their scope and experimental design and are unable to offer broad conclusions about the foraging dynamics of neighboring honey bee colonies.

**TABLE 1:** Comparison of studies investigating spatial foraging patterns of colocalized honey bee colonies.

Sites	Colonies per site	Study period	Sample size	Analysis type	Food type	Food quality
Current study						
3	3	18 April—31 October 2018 10 April—15 October 2019	8049 dances from 230 foraging days	Probabilistic mapping with quantitative and qualitative analyses	Not reported	Not reported
Beekman et al. (2004)						
1	4	23 July—2 August 1999	2885 dances from 6 foraging days	Deterministic mapping with quantitative and qualitative analyses	Specified	Percent sugar in nectar
Waddington et al. (1994)						
2	2	14–17 March 1989 19–22 February 1991	852 dances from 8 foraging days	Deterministic mapping with qualitative analysis	Specified	Not reported

In this study, we analyzed 8049 waggle dances from nine colonies across three landscapes, with three colocalized colonies per landscape, across two complete foraging seasons (April–October, 2018–2019, Table 1) to determine foraging dynamics in respect to those of neighboring colonies. In particular, we used k‐nearest neighbor and k‐means cluster analyses to (1) compare the spatial distribution of foraging among neighboring colonies, (2) identify waggle dance communicated foraging clusters, (3) quantify foraging overlap among neighboring colonies within those clusters, and (4) determine whether and how often neighboring colonies establish single‐colony clusters (defined here as a foraging cluster comprised entirely of dances from the same colony). In doing so, we explore whether neighboring honey bee colonies partition their foraging (and if so, at what scale) or if they do not partition the landscape, which might result in convergence on the same high‐quality patches.

## 2 Materials and Methods

### 2.1 Study Organism

**FIGURE 1:**
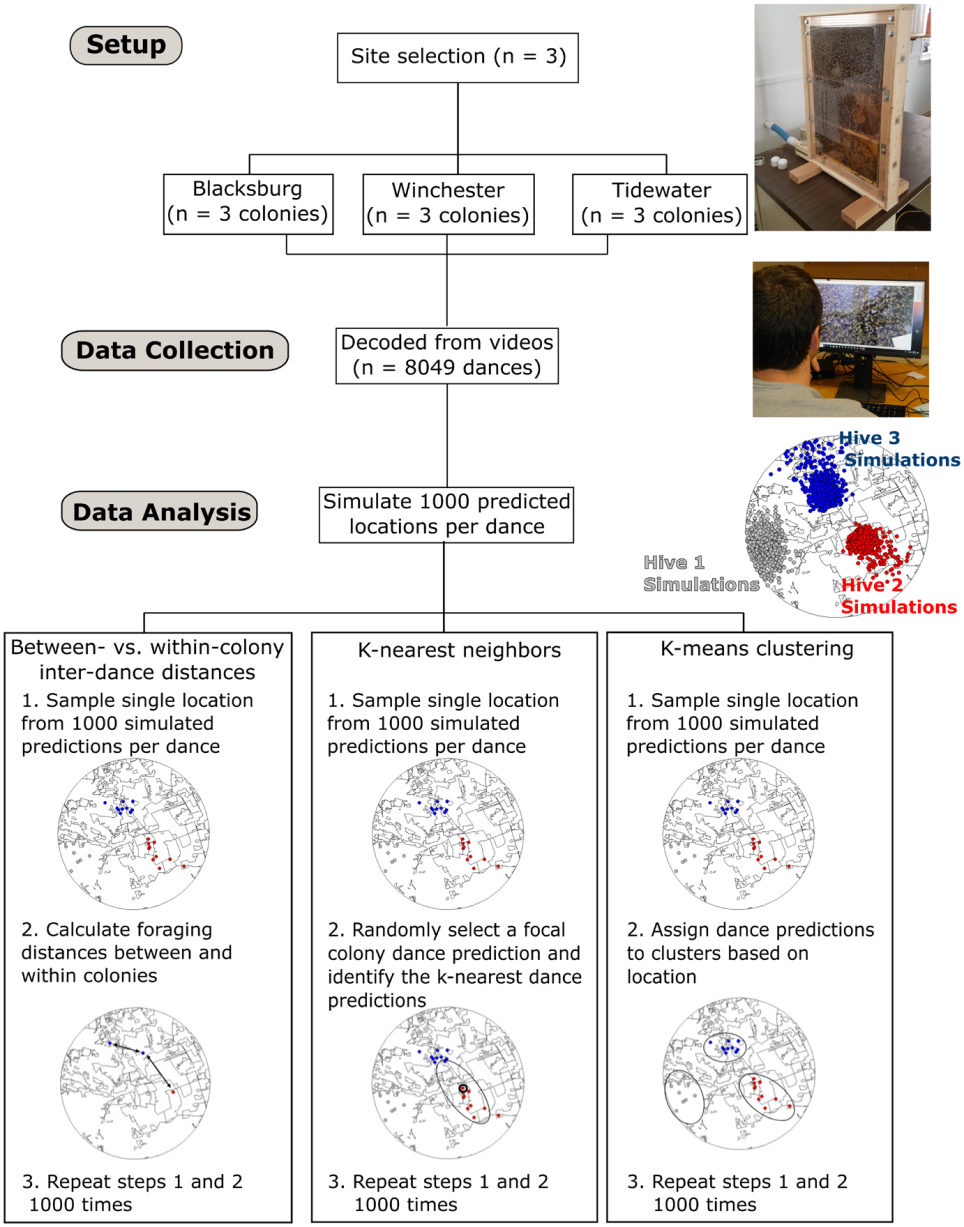
Schematic summarizing the study setup, data collection, and data analysis. Study setup—Nine colonies (three colocalized per landscape) were housed in observation hives across three landscapes in Virginia. Data collection—8,049 dances were recorded while the colonies foraged freely. Data analysis—Foraging spatial patterns were examined using between‐ and within‐colony interdance distance calculations, k‐nearest neighbor analysis, and *k*‐means clustering.

We studied nine honey bee colonies, consisting of a queen and approximately 5000 workers across three different landscapes in Virginia (Figure 1). Colonies were randomly allocated into groups of three adjacent colonies, spaced approximately two meters apart, and maintained within research buildings at the Prices Fork Research Center (PFRC; 37.21148° North, 80.48935° West) in Blacksburg, VA; the Tidewater Agricultural Research and Extension Center (TAREC; 36.66447° North, 76.73278° West) in Suffolk, Virginia; and the Alson H. Smith Jr. Agricultural Research Center (WAREC; 39.11349° North, 78.28449° West) in Winchester, Virginia. Each colony was housed in a glass‐walled observation hive comprised of three vertically arranged, American Standard Deep Langstroth frames, which provided a clear view of the colonies' waggle dances (Figure 1). We installed plumb lines on each hive, which served as a vertical reference for dance decoding. The plumb lines consisted of several weighted fishing lines, spaced 5 cm apart, and hung across the top of the observation hives. The colonies were able to forage freely via a 5 cm × 30 cm PVC piping that extended from the hive to the outside. Importantly, the three landscapes provided distinct ecological conditions for the foraging bees: TAREC consisted primarily of row croplands (Silliman et al. 2022), WAREC of orchard croplands (Steele et al. 2022), and PFRC of a diverse mix of developed, agricultural, and seminatural lands (Ohlinger et al. 2024).

### 2.2 Data Collection

We video recorded waggle dances for 1 h per day 3–5 times per week (weather permitting) at 30 fps using a Canon Vixia HF R82. Waggle dances were typically recorded from 10 to 11 am, but they were always recorded between 9:30 am and 1:30 pm. A wood partition in the observation hive design encouraged the returning foragers to dance on the camera facing side of the hive. We focused the video cameras on a 25 × 20 cm area of high dance activity on each colony. The date, colony ID, time, temperature, and weather were displayed on a datasheet at the beginning of each video. The study period spanned from 18 April to 31 October in 2018 and 10 April to 15 October in 2019, comprising most of the honey bee foraging season for 2 years. We saved the video recordings to SD cards in the field and later uploaded them to a Google Team Drive for analysis in the lab.

### 2.3 Data Collection—Waggle Dance Decoding

We later downloaded the video recordings from the Google Team Drive and converted them into AVI files using FFMPEG on Ubuntu (v. 2004.2021.222.0) and then imported them into ImageJ (version 147 1.52i) for dance decoding using the protocol developed by Couvillon et al. (2012). It was important to calculate the angle offset using the plumb lines because honey bees use gravity to orient the angular component of their dances (von Frisch 1967), and the cameras were not always level during recording. We looked for the first waggle dance and then proceeded through the video by decoding batches of simultaneously dancing bees (Figure 1). To decrease the probability of resampling the same waggle dances, we skipped ahead 6 min after each decoded dance batch before decoding another. We were able to easily identify dancing bees because of their distinct pattern of movement (described above). To decode the dances, we extracted the waggle phase duration, which communicates the distance to the advertised food (von Frisch 1967; Schürch et al. 2013), and the waggle phase angle, which communicates the direction from the colony to the food (von Frisch 1967). We calculated the waggle phase duration as the difference between its start and end. Additionally, we calculated the waggle phase angle by drawing a line along the path from the center of the dancing bees' thorax at its start and end. We then added this angle to the angle offset to correct for the camera's position. Dancing bees perform between 1 and 100+ waggle phases in a single dance (Seeley et al. 2000); however, we averaged the decoded waggle phase duration and angles from a subset of four non‐first, non‐last waggle phases. By doing so, we were able to efficiently extract average duration and direction values that correlate with entire dance averages (Couvillon et al. 2012). In using this method, we decoded numerous waggle dances per foraging day per colony per location (PFRC: 17.4, TAREC: 12.5, WAREC: 17.0).

Waggle dance communications are imprecise: the angular and durational components vary across successive waggle phases in a single dance (Couvillon et al. 2012), and across dances for the same location by different bees (Schürch et al. 2016). Therefore, we plotted waggle dance locations as probability distributions with dispersion parameters that accurately reflect the imprecision of the waggle dance (Schürch et al. 2013, 2019). To do this, we used repeated Monte Carlo sampling from a universal calibration model to simulate the distance component of the dance, and from a von Mises distribution with an appropriate concentration parameter (*k* = 24.5) to simulate the angular component of the dance (Schürch et al. 2019). We used this method to plot 1000 possible locations for each dance (*n* = 8049, Figure 1).

These dances were filmed and decoded as a part of another, larger project to investigate honey bee foraging across three landscapes in Virginia (Ohlinger et al. 2022, 2024; Silliman et al. 2022; Steele et al. 2022). However, as part of that work, we generated large datasets of dances that are published and available to the public through the Virginia Tech Data Repository (PFRC: https://doi.org/10.7294/24139578; TAREC: https://doi.org/10.7294/19755016; WAREC: https://doi.org/10.7294/19952654). For our study here, we reanalyzed the dance data to answer our current objectives.

### 2.4 Statistical Analysis

#### 2.4.1 Calculating Between‐ Versus Within‐Colony Interdance Distances

After plotting the dances, we examined foraging locations advertised by waggle dances on individual days, which was appropriate because honey bee foraging patches change continuously throughout the foraging season (Visscher and Seeley 1982; Waddington et al. 1994; Couvillon et al. 2014). Specifically, we investigated the spatial distribution of foraging locations advertised by the same and different colonies by, first, collecting a Monte Carlo sample consisting of a single simulated location for each dance out of 1000 possible locations per dance (Figure 1). Next, at each site, we randomly selected three simulated dance locations from the Monte Carlo sample per foraging day: a focal dance location, a different dance location from the focal dance colony (within‐colony dance), and a dance location from a nonfocal dance colony (between‐colony dance). We then used the pointDistance() function from the “raster” R package to calculate the distance between the focal and within‐colony dances (the within‐colony interdance distance) as well as the distance between the focal and between‐colony dances (the between‐colony inter‐dance distance). Finally, we calculated the mean value for the between‐colony inter‐dance distance, the within‐colony inter‐dance distance, and the difference of the two across all foraging days in the Monte Carlo sample. We then repeated this process 1000 times to calculate point estimates and 95% confidence intervals for the mean between‐ and within‐colony inter‐dance distances at the three field sites and the mean difference between these values (Figure 1). A positive difference will demonstrate that between‐colony distances are larger than within‐colony distances, whereas a negative difference would demonstrate the opposite. Significance occurs when 95% confidence intervals for the mean difference values are distributed entirely above (greater between‐colony distances) or below (greater within‐colony distances) zero.

#### 2.4.2 Investigating Foraging Overlap Using k‐Nearest Neighbor Analysis

We further investigated the spatial distribution of the dances using k‐nearest neighbor analysis (Figure 1). For each foraging day at all three field sites (PFRC = 71 days, WAREC = 76 days, TAREC = 83 days), we randomly selected a dance location (focal dance location). We then calculated interdance distance values between the focal dance location and all other dance locations to identify the k‐nearest neighbors, with k being one less than the number of dances from the focal dance colony on the foraging day (k = # focal colony dances–1). At each site, we analyzed 1000 Monte Carlo samples, each consisting of a single, randomly selected, simulated foraging location per dance for each foraging day. In particular, we calculated the mean proportion of the k‐nearest dances that were from the same colony (neighboring values) across all foraging days for each Monte Carlo sample at all three field sites. We used this process to construct point estimates and 95% confidence intervals for the neighboring values. For this analysis, we viewed perfect clustering as instances in which 100% of the k‐nearest neighbor dance locations were from the focal dance colony (neighboring value = 1). The neighboring values were additionally converted to a neighboring ratio, which was the quotient of the neighboring value and the proportional abundance of dances from the focal dance colony in the day's dance location sample. 95% confidence intervals for neighboring ratio values that were distributed entirely above 1 indicated that dance locations from individual colonies were nonrandomly clustered.

#### 2.4.3 Investigating Foraging Overlap Using k‐Means Cluster Analysis

Lastly, we performed k‐means cluster analysis to assign the simulated dances locations into groups based on their spatial position in the landscape (Figure 1). Specifically, we used the kmeans() function from the “stats” R package, which applied the algorithm developed by Hartigan and Wong (1979). The optimal number of clusters (*k*) for each kmeans() run was selected using the elbow method, in which single clusters were added until the total within‐cluster sum of squares error (SSE) increased rather than decreased (i.e., the “elbow”). In other words, we selected the highest pre‐elbow *k* value as the optimal number of clusters. As described above, we analyzed 1000 Monte Carlo samples, each consisting of a single, randomly selected, simulated foraging location per dance for each foraging day across the three sites. To account for the variable number of dances per colony on foraging days, we randomly selected for analysis only as many dances (each simulated 1000 times) per colony and foraging day as were available for the colocalized colony with the smallest number of dances. In each sample, we calculated the proportional abundance of dances from each colony in the clusters and identified the most abundant colony within each cluster (dominant colony). We then created 95% confidence intervals at each site by calculating the mean proportional abundance of dances from the dominant colony within clusters (clustering values) across all foraging days in each Monte Carlo sample. Ninety‐five percent confidence intervals for clustering values that were distributed entirely above 0.5 indicated that dance locations from individual colonies made up a significant majority of clusters, suggesting nonrandom within‐colony clustering. For this analysis, perfect clustering occurred where all dance locations in a cluster were from the same colony (clustering value = 1). We define such clusters as single‐colony clusters. After identifying the single‐colony clusters, we calculated the daily mean proportion of clusters that were single‐colony clusters (proportion single‐colony clusters) and the daily mean proportion of colonies that established at least one single‐colony cluster (proportion territorial colonies).

Finally, as part of the cluster analysis, we calculated point estimates and 95% confidence intervals for the maximum distance between dances within the same cluster (cluster size), the minimum distance between dances from different clusters (inter‐cluster distance), and the mean difference between these values. A negative difference will demonstrate that the cluster sizes were smaller than the inter‐cluster distances, whereas a positive difference would demonstrate the opposite. Significance occurs when 95% confidence intervals for the mean difference values are distributed entirely below (greater inter‐cluster distances) or above zero (greater cluster sizes). Although this last analysis does not allow us to resolve between scenarios (see below), it does help us better understand the spatial scale of foraging patterns in the landscape.

### 2.5 Honey Bee Foraging Pattern Scenarios

**FIGURE 2:**
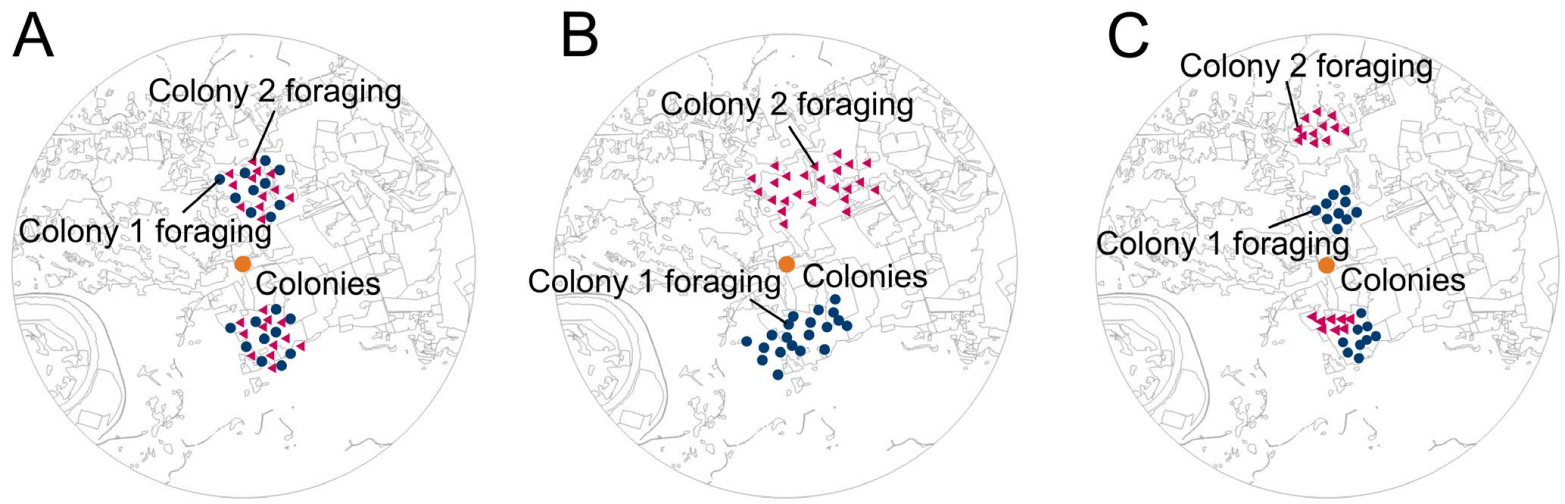
Diagram showing three scenarios for the spatial distribution of dance locations advertised by colocalized colonies. (A) Scenario A—No partitioning, (B) Scenario B—Partitioning on a landscape scale, (C) Scenario C—Partitioning on a local scale, with partitioning on different or the same patches depicted at the top and bottom of the map, respectively. The location of the experimental colonies is symbolized as an orange circle. Dance locations advertised by Colony 1 are depicted as blue circles, while the dance locations from Colony 2 are red triangles.

We hypothesized three possible foraging scenarios for the spatial distribution of dance locations communicated by the neighboring colonies (see Figure 2):

- *Scenario A—Colonies do not partition*. In this case, we expect similar spacing between dances from different and same colonies (i.e., no significant difference in the mean between‐ and within‐colony inter‐dance distances), dances from the focal colony to not be overrepresented as nearest neighbors (neighboring ratios to span across 1), and k‐means clusters to not consist predominately or entirely of dances from the same colony (clustering value CIs span across 0.5).
- *Scenario B—Colonies partition on a landscape scale*. In this case, we expect dance locations advertised by the same colony to be in closer proximity than those from different colonies (significant difference in the mean between‐ and within‐colony inter‐dance distances), dances from the focal colony to be overrepresented as nearest neighbors (neighboring ratios confidence intervals entirely above 1), and k‐means clusters to consist predominately, or entirely, of dances from the same colony (clustering value CIs entirely above 0.5).
- *Scenario C—Colonies partition on a local scale*. In this case, we expect the comparison of between‐ versus within‐colony inter‐dance distances to be noninformative, where it could be that there is either no difference or that between‐colony interdance distances are greater than within‐colony interdance distances. We also expect dances from the focal colony to not be overrepresented as nearest neighbors (neighboring ratios confidence intervals span 1), and k‐means clusters to consist predominantly, or entirely, of dances from the same colony (clustering value CIs entirely above 0.5).

## 3 Results

### 3.1 No Significant Difference in Between‐Colony Versus Within‐Colony Inter‐Dance Distances

**FIGURE 3:**
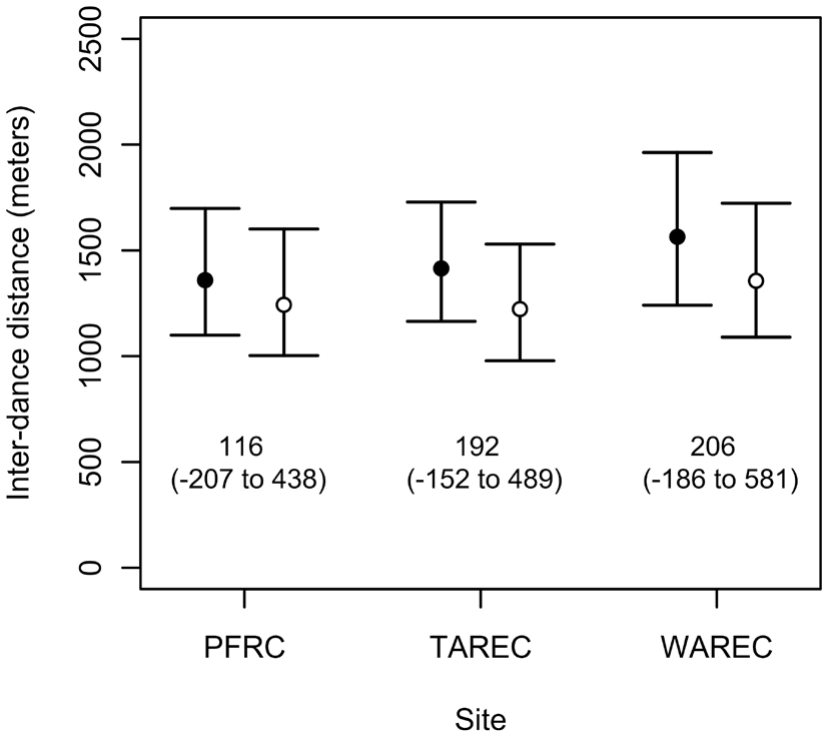
Dance locations advertised by different colonies (black points) were as close to each other as dance locations advertised by the same colony (white points), although there was a numerical trend for between‐colony distances to be greater than within‐colony distances. Error bars represent the 95% confidence intervals for the interdance distance values.

Dances locations advertised by the same and by different colonies were similarly distributed at PFRC (mean interdance distance: between colony = 1359.2 m, 95% CI [1099.2 m, 1697.7 m]; within colony = 1242.8 m, 95% CI [1003 m, 1600.7 m]; mean difference = 115.8 m, 95% CI [−207.5 m, 437.6 m]; *p* = 0.491), TAREC (mean inter‐dance distance: between colony = 1414.7 m, 95% CI [1164.8 m, 1728 m]; within colony = 1222.6 m, 95% CI [978.1 m, 1529.1 m]; mean difference 192.5 m, 95% CI [−152.5 m, 489.2 m]; *p* = 0.242) and WAREC (mean inter‐dance distance: between colony = 1563.8 m, 95% CI [1240.9 m, 1962.4 m]; within colony = 1356.1 m, 95% CI [1090 m, 1722.7 m]; mean difference = 206.1 m, 95% CI [−185.7 m, 581.5 m]; *p* = 0.296; Figure 3). In other words, the relative spatial distribution of dances from either the same or from different colonies was not itself different. However, the point estimates for the mean difference calculated from the between‐ minus within‐colony inter‐dance distances were above 0 at all three sites. This result eliminates Scenario B and supports both Scenario A and C (Figure 2), although Scenario C is more likely because of the above (nonsignificant) zero difference at all three locations (Figure 3).

### 3.2 Neighboring Dance Locations Were Not Disproportionately From the Focal Colony

**FIGURE 4:**
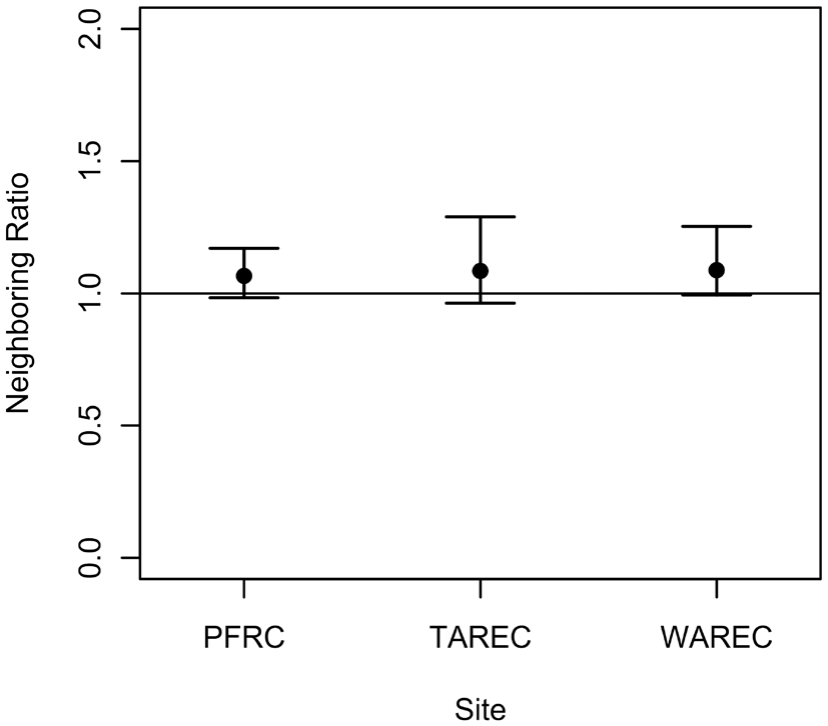
At all three sites, neighboring dance locations were not disproportionately advertised by the same colony. Black points symbolize the point estimates for the neighboring ratios from the k‐nearest neighbor analysis. Error bars represent the 95% confidence intervals for the neighboring ratios. The dashed line reflects our baseline expectation for neighboring ratios under random foraging.

Dance locations from the focal colony comprised a significant majority of its k neighboring dance locations at TAREC (mean daily neighboring value: 0.57, 95% [0.52, 0.61]; *p* = 0.007) and WAREC (0.55, 95% CI [0.5034, 0.58]; *p* = 0.023), but not at PFRC (mean daily neighboring value: 0.52, 95% CI [0.48, 0.56]; *p* = 0.212). The neighboring ratios, which corrected for the proportional abundance of the focal colony, suggest a random distribution of dance locations from the same and different colonies at TAREC (mean daily neighboring ratio: 1.08, 95% CI [0.96, 1.29]; *p* = 0.314), WAREC (mean daily neighboring ratio: 1.09, 95% CI [0.99, 1.25]; *p* = 0.187) and PFRC (mean daily neighboring ratio: 1.07, 95% CI [0.98, 1.17]; *p* = 0.169; Figure 4). This result also eliminates Scenario B but supports Scenarios A and C.

### 3.3 Dance Locations Advertised by Same Colony Aggregate Into Localized Clusters and Single‐Colony Clusters, Likely on Different Flower Patches

**FIGURE 5:**
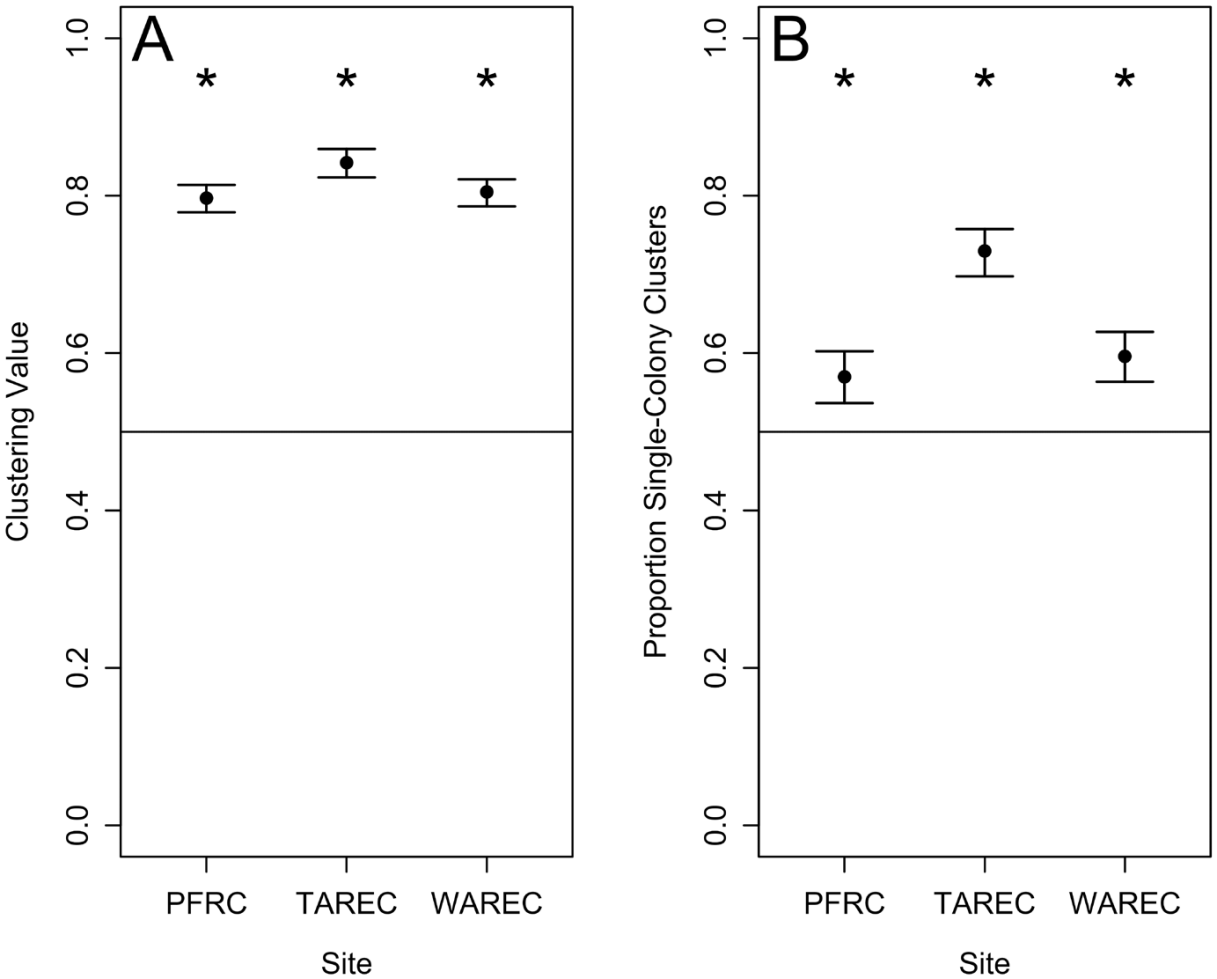
(A) Dance locations advertised by the same colonies aggregated into localized clusters. (B) Single‐colony clusters made up a significant majority of clusters on foraging days. Black points symbolize the point estimates for the mean daily clustering values (A) and daily proportion single‐colony clusters (B). Error bars represent the 95% confidence intervals for these outcomes. The horizontal dashed line at 0.50 is used as a reference to test whether dances from the dominant colony comprise a significant (*) majority of the dances within clusters (A) and whether a significant majority of clusters were single‐colony clusters on foraging days.

Dance locations from the same colony comprised a significant majority of locations within clusters at all three sites (clustering values: PFRC = 0.80, 95% CI [0.78, 0.81], *p* < 0.001; TAREC = 0.84, 95% CI [0.82, 0.86], *p* < 0.001; WAREC = 0.80, 95% CI [0.79, 0.82], *p* < 0.001; Figure 5A). In fact, on each foraging day, most colonies established at least one single‐colony cluster (proportion territorial colonies: PFRC = 0.89, 95% [0.85, 0.94], *p* < 0.001; TAREC = 0.95, 95% CI [0.91, 0.97], *p* < 0.001; WAREC = 0.89, 95% CI [0.85, 0.93], *p* < 0.001). As a result, a significant majority of foraging clusters were single‐colony clusters at all three sites (proportion single‐colony clusters: PFRC = 0.57, 95% CI [0.54, 0.60], *p* < 0.001; TAREC = 0.73, 95% CI [0.70, 0.76], *p* < 0.001; WAREC 0.60, 95% CI [0.56, 0.63], *p* < 0.001; Figure 5B). This result eliminates Scenario A and supports Scenarios B and C.

**FIGURE 6:**
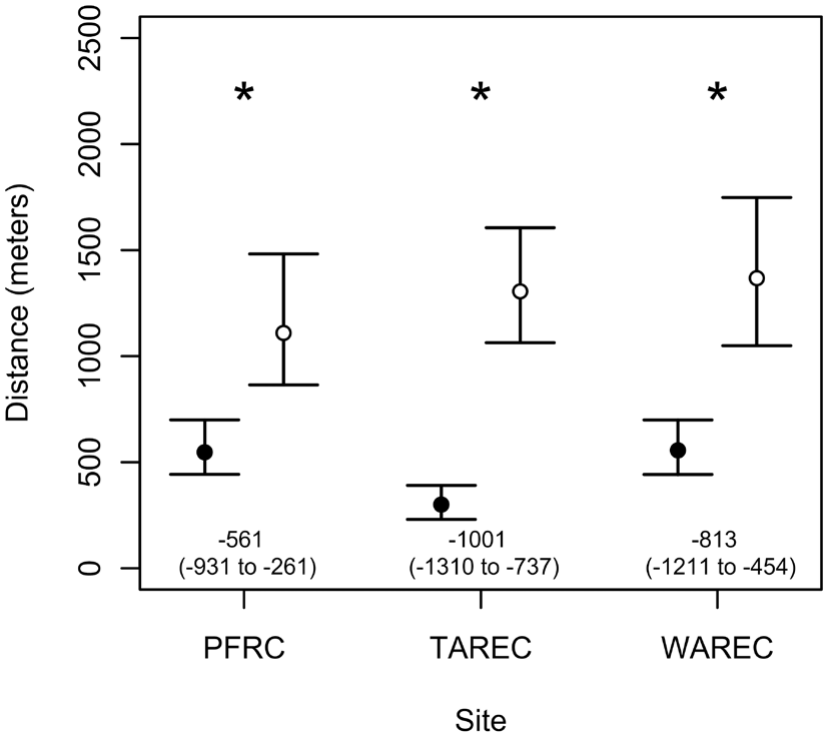
The maximum distance between dances within clusters (cluster size) was significantly (*) lower than the minimum distance between dances from different clusters (intercluster distance). Black points symbolize the point estimates for the cluster sizes. White points symbolize the point estimates for the intercluster distances. Error bars represent the 95% confidence intervals for these values.

Finally, dance locations aggregated into clusters that were smaller (cluster size: PFRC = 547.3 m, 95% CI [442.8 m, 699.6 m]; TAREC = 300.7 m, 95% CI [230.7 m, 391.1 m]; WAREC = 556.3 m, 95% CI [442.3 m, 699.3 m]) than the intercluster distances (PFRC = 1109.3 m, 95% CI [864.5 m, 1481.6 m]; TAREC = 1305.4 m, 95% CI [1063.5 m, 1605.3 m]; WAREC = 1367.6 m, 95% CI [1049.3 m, 1748.1 m]) at all three sites (mean difference: PFRC = −561.1 m, 95% CI [−931.2 m, −260.5 m], *p* = 0.001; TAREC = −1000.7 m, 95% CI [−1310.1 m, −736.7 m], *p* < 0.001; WAREC = −813.2 m, 95% CI [−1210.9 m, −454 m], *p* < 0.001; Figure 6). This suggests that we are more likely to find a spatial pattern represented by the top red/blue pairs in Scenario C (Figure 2) in contrast to the bottom red/blue pairs, suggesting that the partitioning occurs on a local scale, but still on different flower patches.

### 3.4 Colocalized Honey Bee Colonies Display Diverse Spatial Foraging Patterns

**FIGURE 7:**
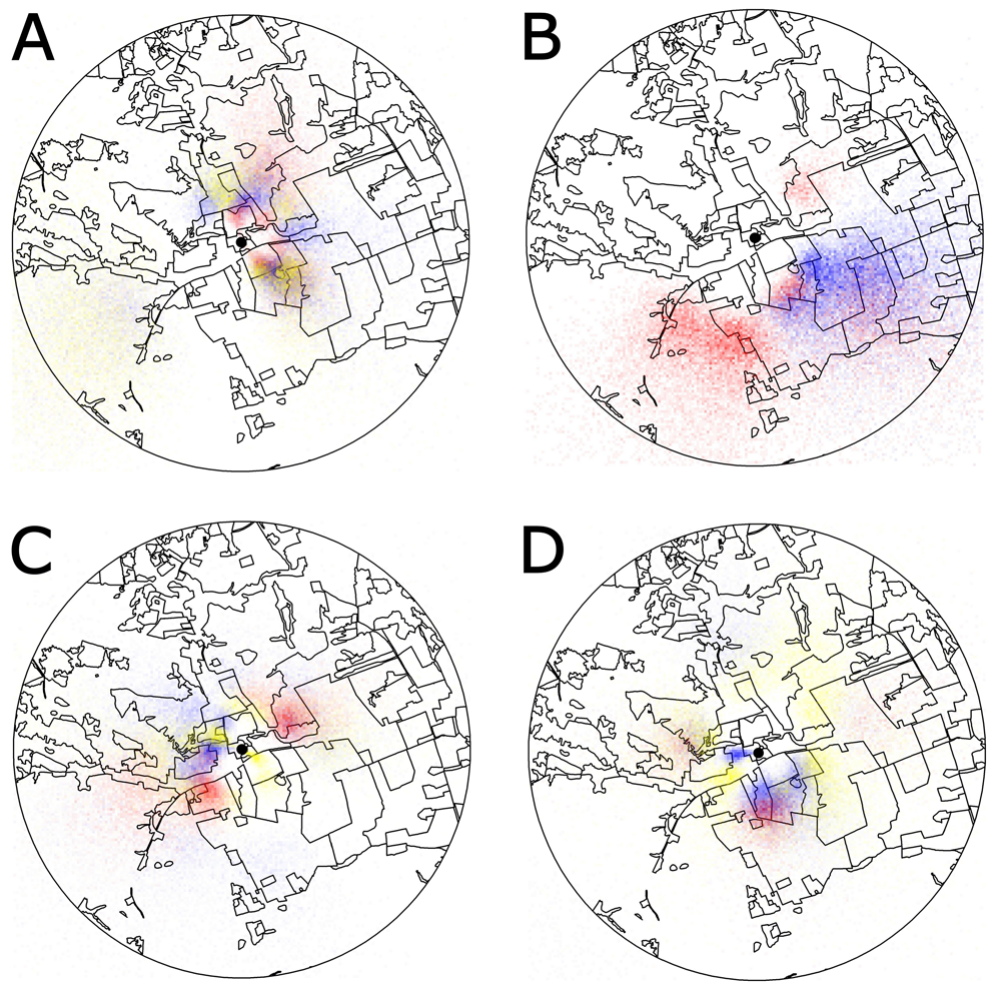
Dance communicated foraging heat maps with opacity denoting the foraging probabilities (low opacity = low visitation and high opacity = high visitation) of three colocalized colonies (colony 1 = red, colony 2 = blue, colony 3 = yellow) within a 2.25 km radius of PFRC. Colocalized colonies (A) did not partition on 12 September 2019, (B) partitioned the landscape into two large foraging regions on 18 June 2018, (C) partitioned the landscape locally across different patches on 29 May 2018, and (D) partitioned the landscape within a patch to the west of the colony on 26 April 2018. Colonies were located in a research building denoted by black points in all four maps.

Qualitatively, dance communicated foraging maps revealed that honey bees displayed spatial foraging patterns consistent with all three scenarios on different foraging days. For example, at PFRC, on 12 September 2019, three colocalized honey bee colonies converged their foraging on large areas north and southeast of the field site (Scenario A; Figure 7A). On 18 June 2018, colonies foraged in two large, multipatch aggregations, with colony 1 foraging to the southwest and colony 2 foraging to the southeast (Scenario B; Figure 7B). Colonies established localized foraging aggregations on multiple different patches on 29 May 2018: colony 1 foraged on patches directly northeast and southwest of the field site, colony 2 foraged on a patch to the west, and colony 3 foraged on patches to the northwest and southeast (Scenario C; Figure 7C). Finally, on 26 April 2018, partitioning occurred within a patch to the west of the field site, with colonies 2 and 3 exploiting the northern and southern portions of the patch, respectively (also Scenario C; Figure 7D). Importantly, while we observed these patterns across different foraging days, localized partitioning on different patches (Scenario C) was most common (see maps for all foraging days at PFRC in the data repository: https://doi.org/10.7294/26276062).

## 4 Discussion

Here, we analyzed spatial patterns in waggle dance communicated foraging in groups of three colocalized colonies replicated across three different landscapes. We showed that distances between dance locations communicated by the same and by different colonies did not differ (Figure 3). Importantly, there was a numerical trend for higher between‐colony interdance distances, which is expected with partitioning by colonies at the local scale. Second, we found in our nearest neighbor analysis that dances from the same colony were not disproportionately represented as neighboring dance locations (Figure 4). Third, k‐means cluster analysis found that dance locations advertised by the same colony disproportionately aggregated into clusters (Figure 5A), often single‐colony clusters (Figure 5B). Finally, we report that the intercluster distances were greater than the cluster sizes at all three sites, which is consistent with localized across‐patch partitioning over localized within‐patch partitioning. In summary, although honey bees occasionally forage with all scenarios (Figure 7), our most commonly observed pattern supported by the most evidence demonstrates that honey bees locally partition their foraging (Scenario C), producing an emergent pattern that may allow neighboring colonies to limit competition by dividing up patches at a local level.

The honey bee foraging process has adapted to flexibly exploit resources according to both the availability of quality resources in their environment (T. D. Seeley 1986, 1994; Seeley et al. 1991) and colony nutritional demands (T. D. Seeley 1989; Camazine 1993; Dreller et al. 1999; Dreller and Tarpy 2000). Importantly, this process involves both scouting, which is a random search behavior for finding new resources (Biesmeijer and Seeley 2005), and recruitment, which concentrates foraging on valuable resources (von Frisch 1967; T. Seeley 1995). Together, these processes allow honey bees to consistently identify and exploit even small patches of quality resources over a large area (T. D. Seeley 1987). As a result, the honey bee foraging system is thought to be an optimal foraging strategy that maximizes the net energetic efficiency of resource collection (Schmid‐Hempel et al. 1985; T. D. Seeley 1994). Therefore, we expect that honey bee colonies located in proximity to each other will similarly assess and exploit the same resource patches. Alternatively, within‐patch competition might deplete the quality of resources, which could influence foraging decisions and potentially lead to resource partitioning, either among colonies or among cohorts of workers from each colony.

Previous waggle dance decoding studies report that neighboring colonies often exploit different foraging patches, suggesting that colocalized colonies might partition their foraging (Waddington et al. 1994; Beekman et al. 2004). However, these studies investigated foraging of paired colonies, positioned in only one (Beekman et al. 2004) or two (Waddington et al. 1994) landscapes, over short time periods (Table 1). Additionally, they used analytic approaches that do not reflect the imprecision inherent in the dance communication (Couvillon et al. 2012; Schürch et al. 2013, 2016, 2019). In this study, we address these limitations by using a probabilistic approach to waggle dance mapping to investigate spatial patterns in waggle dance communicated foraging across sites. In particular, we analyzed waggle dance communicated foraging of three colonies located in each of three landscapes for two entire foraging seasons (April–October), which generated a large dataset. In doing so, we confirm the resource partitioning reported in previous studies across our three field sites, while uncovering consistent landscape‐ and local‐scale patterns across the sites. In other words, we demonstrated that dances from the same colony were nonrandomly aggregated in foraging clusters at one level. However, at another level, the relative positions of the clusters within the broader landscape were independent of their colony origin (Scenario C, Figure 2C). While we did observe some foraging overlap between neighboring colonies, our results suggest that colonies often established single‐colony clusters: 62% of clusters across the three field sites were single‐colony clusters and, on average, 91% of colonies established at least one single‐colony cluster on days when they were actively foraging.

How might this spatial pattern fit into an optimal foraging strategy? Importantly, foraging experiments have demonstrated that competition influences foraging by depleting resource values (exploitative competition; Balfour et al. 2013, 2015) and by increasing the costs of extraction through direct interactions among competitors (interference competition; Nagamitsu and Inoue 1997). For example, honey bee foragers are deterred from lavender via bumble bee‐imposed exploitative forces (Balfour et al. 2013; Balfour et al. 2015), and various social bees' aggressive foraging produces a foraging dominance hierarchy among social bees through interference competition (Nagamitsu and Inoue 1997). Therefore, the foraging activity of competitors is a relevant factor, both indirectly and directly, in foraging currency calculations (Milinski 1982; Balfour et al. 2013, 2015). Of course, here we are talking about within‐species competition, which is still relevant, especially when foraging forces number so many thousands per colony. In our case, the simultaneous, overlapping, landscape‐scale pattern and aggregated local‐scale pattern are consistent with a foraging strategy that selectively exploits the wide range of available resources while decreasing between‐colony competitive forces. Importantly, our study is one of the first to investigate the potential role of intercolony competition on honey bee foraging spatial patterns (Table 1). Therefore, we provide much needed insights into how honey bees, and potentially other social foragers, might respond adaptively to competition with conspecifics.

How might honey bee foraging and recruitment produce this spatial pattern? Critically, the opposing stochastic scouting and the deterministic foraging and recruitment processes may be key features, allowing for the hierarchical spatial pattern with large‐scale dispersion of dance locations among neighboring colonies and small‐scale aggregation of dance locations within colonies. For example, scouts from different colonies may find different patches of the same resources or different resources of comparable quality. In this case, each of the colonies then concentrates its foraging on the best among the identified options through selective individual foraging and recruitment (T. D. Seeley 1986, 1994, 1995; Seeley et al. 1991). Scouts might occasionally converge on the same patches; however, each colony is likely to find these patches at different points in time. As a result, foragers from each colony may experience different levels of competition and resource quality/availability in the patches depending on their order of arrival at the patch. Each colony may then decrease competition with other colonies by exploiting several different high‐quality patches that are sometimes located in close proximity (i.e., partitioning the landscape locally). Future studies can test this possibility by integrating key features from previous studies, such as food‐specific mapping (Waddington et al. 1994; Beekman et al. 2004) and nectar sugar concentration measurements (Waddington et al. 1994), with our comprehensive study design and probabilistic mapping methodology (Table 1).

Although we did not directly test the influences of competition on the spatial foraging patterns communicated by colocalized colonies, the patterns that we did observe are consistent with OFT predictions. In particular, our results strongly suggest that the honey bee foraging system produces an emergent foraging pattern that reduces both within and among colony competition by establishing foraging aggregations that partition the landscape locally, probably across different flower patches. In doing so, these results provide relevant insights into the potential complementary roles of stochastic scouting and deterministic foraging and recruitment processes for efficient social foraging.

## Author Contributions

**Bradley D. Ohlinger:** conceptualization (equal), data curation (lead), formal analysis (lead), investigation (equal), methodology (equal), software (lead), validation (lead), visualization (lead), writing – original draft (lead), writing – review and editing (equal). **Margaret J. Couvillon:** conceptualization (equal), funding acquisition (equal), investigation (equal), methodology (equal), project administration (equal), supervision (supporting), writing – review and editing (equal). **Laurence W. Carstensen:** conceptualization (supporting), formal analysis (supporting), writing – review and editing (supporting). **Roger Schürch:** conceptualization (equal), data curation (supporting), formal analysis (supporting), funding acquisition (equal), investigation (equal), methodology (equal), project administration (equal), software (supporting), supervision (lead), validation (supporting), visualization (supporting), writing – review and editing (equal).

## Conflicts of Interest

The authors declare no conflicts of interest.

## Data Availability

All data generated and analyzed for this study are included in a public data repository (https://doi.org/10.7294/26276062).
